# Supplementary material for: Are carbon emissions trading and green financial instruments synergistic? -Comprehensive quantitative research based on content analysis
Source: PLoS One. 2024 Mar 7;19(3):e0298601. doi: 10.1371/journal.pone.0298601 (PMC10919601; doi:10.1371/journal.pone.0298601)
Supplement: S1 Data — (ZIP) [file pone.0298601.s001.zip › Data/地级市Z_F工作报告.zip!/░▓╗╒/═¡┴Ω╩╨/└┤╫╘╣1⁄2╓┌║┼í╛┬φ┐╦╩2╛▌═°í┐_2018.docx]

各位代表：

　　现在，我代表铜陵市人民政府，向大会报告工作，请予审议，并请市政协委员和其他列席人员提出意见和建议。

　　一、过去五年工作回顾

　　过去的五年，是铜陵发展进程中极不平凡的五年。面对经济发展新常态、城市转型新关口，全市人民深入贯彻落实党的十八大、十九大精神，坚持以习近平新时代中国特色社会主义思想为指导，在省委、省政府和中共铜陵市委的坚强领导下，坚持稳中求进工作总基调，迎难而上、砥砺奋进，较好地完成了市十五届人大历次会议确定的目标任务，经济社会发展取得了令人鼓舞的成就。

　　这五年，铜陵发展能级显著提升。地区生产总值跨过千亿元台阶，年均增长9.5%，服务业增加值占生产总值比重由24.4%提高到33%。财政收支双双超过160亿元。规上工业主营业务收入突破3500亿元，是2012年的1.6倍；累计增加规上工业企业166家，增长四成以上。五年开工亿元以上项目601个，竣工511个，完成投资近2400亿元。开发园区规上工业产值、财政收入等主要经济指标实现倍增。铜陵港跻身亿吨大港，吞吐量是2012年的2倍，成为安徽第二大港。

　　这五年，铜陵发展格局深刻变化。行政区划实现重大调整，跨江发展成为现实，一江两岸呈现协调联动发展新格局。两年来，枞阳区域交通、水利完成投资58.5亿元，是“十二五”的3倍。城乡规划体系不断完善，城市拉开框架，新区拔节生长，建成区面积由71平方公里扩大到81.2平方公里，一批重大基础设施项目相继建成；美丽乡村建设由点向面扩展，建成省级中心村122个、市级中心村140个，城乡面貌发生颠覆性变化。宁安高铁、京福高铁等先后通车，等级公路里程由1233公里增加到1649公里，交通区位条件发生巨变。融入长三角城市群，区域发展地位不断巩固提升。

　　这五年，铜陵发展转型明显提速。坚持创新驱动，新建省级以上研发机构50家，规上工业企业研发平台覆盖率达30%，万人发明专利拥有量由2.04件增加到6.7件。战略性新兴产业产值由385.6亿元增加到960亿元；传统产业改造完成投资1824亿元，年均增长28.4%；铜材加工量突破200万吨，是五年前的3.2倍；新能源汽车、机器人、手机等终端产品从无到有；循环经济升级发展。生态环境整治修复力度前所未有，依法关停、取缔小矿山32家、小选厂（点）251家，拆除小码头和非码头设施167处，节能“双控”全省领跑，万元地区生产总值能耗下降27.7%，主要污染物排放量持续削减，城乡生态环境持续优化。国家节能减排示范市绩效考核连续三年优秀、总评优秀；资源型城市转型成为全国唯一连续四年获得优秀的城市。

　　这五年，铜陵发展活力持续释放。改革全面发力、多点突破、纵深推进。供给侧结构性改革有力有效，先后淘汰落后产能企业52家，2015年以来商品住房去化周期由13个月降到8个月，企业长期杠杆率由69.7%降至66.8%，争取政府债券192亿元。“放管服”改革持续深化，行政许可事项精简14.8%，“多规合一”试点经验在全省推广。财税国资改革不断突破，投融资、公共资源交易、土地管理、征收安置体制机制进一步理顺，农村“三权”分置改革扎实推进。新登记市场主体5.3万户，年均增长19%；新注册商标3265件，年均增长42.6%。对外开放向广度深度拓展，与“一带一路”国家和地区经贸合作持续加强；累计引进亿元以上项目1463个，利用内资2173亿元、年均增长14.3%，利用外资13.7亿美元，新批外资企业44家，开放型经济发展水平明显提高。

　　这五年，铜陵发展成果普惠全民。民生支出占总支出比重达85%，民生工程绩效评价连续五年居全省前列，枞阳县23项社会保障待遇实现提标。脱贫攻坚稳扎稳打、连战连胜，7.53万人告别贫困，枞阳县贫困发生率由13.4%下降为4.8%。3.13万户棚改居民安居圆梦，4.8万被征收户喜迁新居，整治65个老旧小区惠及居民2.6万户，改造农村危房1.31万户，住房公积金制度覆盖面不断扩大。11万农村人口饮水安全问题得到解决。新增就业13.67万人，城乡居民人均可支配收入增速超过经济增速。社会保障由制度全覆盖向人群全覆盖转变，特困人员供养、城乡低保等不断提标。各项社会事业快速发展，社会文明程度显著提升，67人当选“中国好人”，百万人口“中国好人”当选率居全国前列。法治铜陵、健康铜陵、平安铜陵、智慧铜陵建设不断深化，人民群众获得感、幸福感、安全感显著增强。

　　五年来，我们全面加强政府自身建设。依法规范政府决策机制和行政执法行为。自觉接受人大法律监督、工作监督和政协民主监督，办结人大代表建议949件、政协提案1424件。扎实推进政府系统全面从严治党，深入开展党的群众路线教育实践活动、“三严三实”专题教育、“两学一做”学习教育、“讲重作”专题教育，严格执行中央八项规定精神和省、市委相关规定，强化行政监察和审计监督，严肃查处了一批侵害群众利益的“四风”和腐败问题，营造了风清气正、干事创业的良好政治生态。

　　尊敬的各位代表！

　　刚刚过去的一年，我们积极适应把握引领经济发展新常态，以供给侧结构性改革为主线，大力实施五大发展行动计划，扎实开展“产业集聚基地推进年”“特色小镇培育建设年”“协调发展提升年”活动，统筹推进各项工作，经济社会保持平稳健康发展，为本届政府画上了圆满句号。预计，全年实现地区生产总值1150亿元，增长8.1%；完成财政收入167.8亿元，增长9.1%；固定资产投资1330亿元，增长11%；社会消费品零售总额340亿元，增长12.3%；城镇、农村居民人均可支配收入33237元、13139元，分别增长8.5%、9%；节能减排实现年度控制目标。

　　这一年，我们坚持创先争优，成为全国首批资源型城市产业转型升级示范区，蝉联全国文明城市，荣获国家森林城市，再捧全国综治最高奖“长安杯”。一年来，我们主要做了以下工作：

　　第一，千方百计稳定经济增长。扎实推进“五个暖企”，实施“四送一服”双百工程，进一步优化营商环境。投入产业扶持资金3.6亿元，组建9支基金、总规模92.5亿元，推动企业完成直接融资108亿元。全年新开工亿元以上项目145个、竣工110个，完成投资620亿元，增长8.7%。有色“奥炉”改造主体工程完工，泰新新能源汽车整车下线，北大青鸟陶瓷电路板、万华秸秆综合利用、金誉金属电池铝箔等一批产业项目加快推进。亿元以上在建省外投资项目到位资金增长15.9%，实际利用外商直接投资增长15.2%；新引进高层次科技人才团队10个，入围省级团队创历史新高。

　　第二，持续深入调整产业结构。统筹推进“三重一创”、制造强市建设，铜基新材料产业基地产值、投资、税收保持较快增长，4个市级产业基地产值增长20%以上，大气环境综合立体监测产业化项目入列省重大新兴产业工程。战略性新兴产业产值增长31%，居全省第2位。传统产业改造升级持续发力，抓技改促转型典型经验受到省政府通报表扬。服务业发展提速提质，铜官区国家服务业综合改革试点稳步实施，枞阳县国家全域旅游示范区创建全面启动，电子商务进农村实现“三个全覆盖”，限额以上网上商品零售额增长120%，进境水果指定口岸正式运营，皖中南B型保税物流中心申报建设积极推进。培育新型农业经营主体546个，增长44.4%，农业全产业链开发成为全国创新示范区。

　　第三，坚定不移推进改革创新。建立完善科技创新政策体系，设立科技型中小微企业贷款风险补偿资金池，在全省率先开展科技创新券试点。狮子山国家级高新区获国务院批准，国家印制电路板质检中心基本建成。创新建立行政监管清单制度，“九证合一”营业执照全省首发，市场监管“双随机、一公开”联查率先试点，“互联网+政务服务”平台实现省市县（区）互联互通。推进国资国企改革，铜化集团混合所有制改革引入战略投资者，市交通投资公司、普济圩现代农业集团完成组建，盘活国有资产资源54.5亿元。农村“三变”改革试点顺利启动。国有林场改革全面完成。供销社综合改革积极推进。城市管理执法体制改革稳步实施。

　　第四，统筹协调推进城乡建设。交通建设加快推进，庐铜铁路基本建成，池州长江公路大桥及连接线建设进展顺利，G3长江公铁大桥项目前期提速推进，国道347进入路面施工，国道236与省道221、335路基成形，农村道路畅通工程三年计划提前完成。新建改造市政道路10条，新投放清洁能源公交车119辆，建成一批电动汽车充电桩，完成新一轮出租车更新；市档案馆、党史馆、非遗展示馆、数字文化体验馆等全面竣工，市一中西湖校区二期工程、儿童医院主体建成，大铜官山公园建设持续推进。滨江生态岸线北延工程全面完工，大通、横港段岸线整治快速推进。镇村建设成果丰硕，农村环境“三大革命”取得阶段性成效，钟鸣镇成为第二批全国特色小镇，胥坝乡群心村、东联镇合兴村人居环境建设获得全国表彰。水利完成投资14亿元，水利水毁工程全面修复，6个中小河流治理项目完工，21座病险水库除险加固，新建和改造提升排涝泵站7座，城乡防洪排涝能力不断增强。

　　第五，坚持不懈优化生态环境。坚持问题导向，狠抓中办调研回访、中央和省环保督察反馈问题整改，有效解决了一批突出环境问题。推进“蓝天、碧水、净土”工程，全面关停金昌冶炼厂生产系统，淘汰黄标车及老旧车1391辆，整治企业物料堆场14个，秸秆禁烧保持“零火点”。完成黑砂河、滨江花园东侧黑臭水体整治，黑砂河整治工程获得中国人居环境范例奖；彻底消除城市饮用水水源地环境安全隐患，工业集聚区污水处理设施基本实现全覆盖；建立落实四级“河长制”，国控、省控江河湖泊监测断面水质达标率保持100%。我市成为全国环境治理项目推进快，重点区域大气、重点领域水环境质量明显改善的4个城市之一。开展林业增绿增效行动，人工造林9670亩，新增和改造提升绿道69公里、绿化面积258万平方米，治理矿山裸露山体面积1200多亩，促进了城市修补和生态修复。

　　第六，倾心倾力保障改善民生。全面实施脱贫攻坚“十大工程”，建成村级集体光伏电站43个、户用光伏电站7650户，实施特色产业扶贫项目2.6万个，建成就业扶贫工厂和扶贫驿站60个，小额信用贷款发放额增长10倍，创新出台慢性病门诊“195”补充政策，贫困家庭在校学生资助实现全覆盖。实施积极的就业政策，城镇登记失业率3.17%。机关事业单位养老保险统筹并轨，基本医疗保险实现跨省联网直接结算。教育事业协调发展，新建改建公办幼儿园5所，24所薄弱中小学校舍完成改造，县区义务教育均衡发展全部通过国家认定。综合医改持续深化，基层首诊率、家庭医生签约服务覆盖率不断提高。公共文化服务体系示范区创建高水平通过文化部中期督查，《铜文化书系》完成编撰。全面两孩政策有效落实。全国居家和社区养老服务试点工作获民政部、财政部通报表彰。全民健身和竞技体育全面发展，全国运动会实现金牌“零突破”。质量安全、粮食安全、食品药品安全得到巩固。安全生产持续稳定向好。扎实落实十九大维稳安保任务，认真处理群众来信来访，社会大局和谐稳定。社会科学、新闻出版、广播影视、文学艺术、统计、气象、地震、人防、消防、兵役等工作取得新进步，外事侨务、对台、港澳、外宣、民族宗教、妇女儿童、红十字等事业实现新发展。

　　各位代表！

　　回顾过去的五年，全市人民以强烈的使命担当，一往无前的奋斗姿态，抢抓历史机遇，办大事解难事干实事，推进深层次变革，推动全方位发展，先后荣获10多项国家级荣誉，争先实施20多项国字号试点示范，落实上级重大决策多次受到国务院、省政府通报表扬，创造了铜陵发展史上新的辉煌。

　　五年的成就，充分展现了广大干部群众的苦干实干精神和创新创造风采，极大地增强了铜陵人民的自信心和自豪感。今天，我们跨入新时代、立足新起点，更有底气、更有信心，接续开创铜陵“五大发展”的光明前景与美好未来！

　　五年的成就，是习近平新时代中国特色社会主义思想科学指引的结果，是省委、省政府和市委坚强领导的结果，是全市人民共同奋斗的结果。在此，我代表市人民政府，向全市人民，向各民主党派、各人民团体和各界人士，向驻铜解放军指战员、武警官兵和政法公安干警，向关心支持铜陵改革发展的省直各部门、兄弟城市和海内外友好人士，表示最衷心的感谢！

　　尊敬的各位代表！

　　总结回顾过去，我们还必须清醒地认识到前进道路上面临的困难和问题。主要是：经济总量不大且结构性矛盾突出，新旧动能转换不快，自主创新能力不强，发展后劲支撑不足，实体经济发展困难不少，县域经济、园区经济与外向型经济发展不充分。城乡之间、南北之间发展不平衡问题突出，民生以及交通、水利等领域短板不少，脱贫攻坚任务艰巨。生态环境容量压力突出，治理保护任重道远。基本公共服务资源分布不均，供给质量不高。社会矛盾、不稳定因素和潜在风险较多。政府部门及工作人员的理念、能力、作风与形势发展不相适应，“四风”和腐败现象仍然存在。我们务必防骄破满，不等不靠，着力采取措施加以解决。

　　二、今后五年的奋斗目标和主要任务

　　今后五年对铜陵发展至关重要。我们要高举中国特色社会主义伟大旗帜，以习近平新时代中国特色社会主义思想为指导，全面贯彻落实党的十九大精神，按照省、市第十次党代会和市委十届五次全会的部署要求，统筹推进“五位一体”总体布局，协调推进“四个全面”战略布局，坚持以人民为中心的发展思想，以新发展理念统领发展全局，以供给侧结构性改革为主线，以五大发展行动计划为总抓手，坚定实施“七大战略”，抢占竞争制高点，争当发展排头兵，着力打造“六个新铜陵”，努力在转型发展中闯出新路，决战决胜全面建成小康社会，奋力开启现代化幸福美丽新铜陵建设新征程。

　　主要目标是：全面完成市第十次党代会和“十三五”规划目标，高水平建成小康社会。经济保持中高速增长，主要指标总量力争在省内进位次。产业迈向价值链中高端，质量效率全省领先，全要素生产率显著提高，成为国家创新型城市。城乡融合发展体制机制不断健全，一江两岸协调发展，新型城镇化和城乡一体化质量明显提升，常住人口城镇化率达到60%。生态环境质量明显改善，生态文明建设成为全国资源型城市样板。城乡居民收入稳步提高，现行标准下农村贫困人口全部脱贫。全面打好三大攻坚战，改革创新、转型发展、城乡统筹、环境建设、全面小康和现代化建设走在全省前列。

　　（一）坚持创新发展，构建现代产业新体系。健全科技创新“四大体系”，实施高新技术企业培育行动计划，推动规上工业企业研发机构全覆盖，建设具有国际影响力的铜产业研发基地，打造长三角科技成果转化应用基地。强力推进“三重一创”建设，高端化发展铜产业，新型化改造传统产业，规模化培育新兴产业，打造世界级铜产业集群，培育壮大新能源汽车及零部件、智能装备、节能环保等新兴制造业产业集群，推动互联网、大数据、人工智能和实体经济深度融合，争创“中国制造2025”国家级示范区。大力引进和培育高层次人才团队，激发和保护企业家精神，弘扬工匠精神、劳模精神，促进更多社会主体投身创新创业。加快推进经济体制改革，转变政府职能，发展混合所有制经济，壮大民营经济，不断完善公平竞争的市场环境，打造“四最”营商环境。

　　（二）坚持协调发展，开创区域联动新局面。着力推进新型城镇化。全面放开城市落户限制，加快农业转移人口市民化步伐。培育发展区域特色小镇，推动美丽乡村中心村建设全覆盖，促进产城融合、港城融合、城乡融合、南北融合，形成现代城市、特色小镇、美丽乡村协调发展格局。实施乡村振兴战略。坚持农业农村优先发展，巩固和完善农村基本经营制度，构建现代农业“三大体系”，加强农村突出环境问题综合治理，加快推进农业农村现代化，推动农业全面升级、农村全面进步、农民全面发展。改善发展基础条件。对接长江经济带综合立体交通走廊，谋划推动合温铁路、北沿江高铁、合庐铜黄城际、北沿江高速等重大项目，统筹谋划过江通道。升级加密南北区域路网，加大水利、电力、供气、通信等设施建设力度，重点向江北地区倾斜。

　　（三）坚持绿色发展，创建生态文明新样板。加快经济绿色转型步伐。实行更严格的投资项目环境准入制度，深入实施“8311”行动计划，打造循环经济升级版，建设资源型城市产业转型升级示范区。加强环境污染综合治理。开展大气污染防治行动，加快重点行业污染源整治和移动源污染防控，推进秸秆禁烧和综合利用。实施水污染防治工程，基本完成主城区雨污分流改造、黑臭水体治理。推进土壤污染防治，突出冶炼、化工、矿山等重点污染区域治理，完成破坏区域生态修复。加大生态系统保护力度。统筹山水林田湖草系统治理，加强生物多样性保护。突出长江生态大保护，启动沿江重化工业搬迁改造计划。巩固国家森林城市创建成果，加强湿地保护修复，基本建成大铜官山公园，争创国家生态园林城市。

　　（四）坚持开放发展，打造内陆开放新高地。全面加强区域合作，深度参与长三角产业分工协作，构建承接沿海地区产业、资本、技术转移新高地，打造长三角城市群西翼新兴增长极。主动对接合肥都市圈，规划建设江北港新区，推动合铜发展带建设。坚持港城一体发展，优化功能布局，发展江海联运，主动融入国家自由贸易港建设，打造更高能级的对外开放平台。加大“双招双引”力度，优化招商引资方式，强化招才用贤能力，推动开发园区改革创新发展。积极融入“一带一路”倡议，扩大国际经贸和产能合作，提高开放型经济发展质量和水平。

　　（五）坚持共享发展，创造幸福美好新生活。着力保障和改善民生，让改革发展成果更多更公平惠及全体市民。坚决打赢脱贫攻坚战。坚持把提高脱贫质量放在首位，注重扶贫与扶志、扶智相结合，瞄准贫困人口精准帮扶，促进各项扶贫脱贫措施落地见效，确保到2020年全市农村贫困人口全部脱贫、贫困村全部出列。全面提高公共服务水平。深化教育改革，扩大学前教育资源，推进城乡义务教育优质均衡发展，率先实现教育现代化。深化医药卫生体制综合改革，强化“三医联动”，建设健康铜陵。加强就业服务，促进创新创业，建设技工强市。建立多主体供给、多渠道保障、租购并举的住房制度。完善全覆盖、多层次、可持续的社会保障体系，统筹城乡社会救助，发展残疾人事业。切实增强综合治理能力。深化平安铜陵建设，完善社会治理体制，健全公共安全、社会治安防控和社区治理体系，打造共建共治共享的社会治理格局。培育践行社会主义核心价值观，推进“传育立行”教育工程，巩固提升文明城市创建成果，提升铜陵精神海拔。

　　各位代表！

　　未来征途不会平坦，困难挑战无可避免。实现今后五年的奋斗目标，绝不会轻轻松松、舒舒服服。我们一定要进一步凝心聚力，艰苦奋斗，戒骄戒躁，较真碰硬，以时不我待、只争朝夕的精神，奋力走好新时代的长征路，不辱历史使命，不负人民重托！

　　三、2018年主要工作安排

　　今年是全面贯彻落实党的十九大精神的开局之年，是决胜全面建成小康社会、实施“十三五”规划承前启后的关键一年。我们要以习近平新时代中国特色社会主义思想为指导，坚持稳中求进工作总基调，坚持新发展理念，紧扣社会主要矛盾变化，按照高质量发展要求，以供给侧结构性改革为主线，统筹推进稳增长、促改革、调结构、惠民生、防风险各项工作，大力推进改革开放，大力推进创新驱动，推动质量变革、效率变革、动力变革，打好防范化解重大风险、精准脱贫、污染防治三大攻坚战，促进经济社会持续健康较快发展，确保开好局、起好步。

　　今年经济社会发展的主要预期目标是：全市生产总值增长8%；财政收入增长5%以上；固定资产投资增长8.5%；社会消费品零售总额增长10%；城镇常住居民人均可支配收入增长与经济增长同步，农村常住居民人均可支配收入增长8.5%以上，增幅高于城镇常住居民增幅；居民消费价格涨幅3%以下；城镇新增就业2.5万人、调查失业率控制在5%以内；完成省政府下达的节能减排年度目标任务。重点做好七个方面工作：

　　（一）着力发展实体经济，提高经济增长质量

　　进一步优化供给质量。大力破除无效供给，综合整治沿江区域化工企业，淘汰小矿山等落后产能企业9家，鼓励推动企业重组，完成“僵尸企业”处置。大力培育战略性新兴产业，突出“强链、延链、补链”，强化关键共性技术攻关，发展关联配套产业，提高省、市重大基地产业集聚效应。以新技术、新业态和新模式优化升级传统产业，全年实施亿元以上技改项目50个以上。促进重点领域“两化融合”，发展智能制造，建设一批数字化车间和智能工厂，完成200家规上企业两化融合评估诊断。深入开展“增品种、提品质、创品牌”行动，推进商标品牌战略，新增省级新产品10个、工业精品5个以上，争创全国标准化示范市。加快住房制度和长效机制建设，推进商业办公地产去库存，引导商品住房消费，促进房地产市场更加平稳健康发展。

　　全力支持实体经济发展。深化暖企行动，继续开展“四送一服”活动，拓展服务广度和深度，提升企业获得感。全面落实降成本各项措施，扩大直供电、直供气范围，清理规范涉企保证金，进一步降低要素和物流等生产成本，多渠道减轻企业负担。大力发展普惠金融，引导金融机构加大对中小微企业支持力度，完善“税融通”、续贷过桥资金业务，促进金融脱虚向实，新增贷款80亿元。推动多层次资本市场建设，培育上市企业，新增“新三板”挂牌企业3家、省股权交易中心挂牌企业25家，力争完成直接融资100亿元。鼓励各类投资基金进行股权投资，支持企业债券债权融资。坚持阶梯式培育企业，推动“个转企、小升规、规改股、股上市”，新增规上企业45家、限上企业40家。实施“单打冠军”培育专项行动，新增“专精特新”企业20户。

　　推动服务业集聚发展。深入推进铜官区国家服务业综合改革试点，落实三年行动方案，滚动实施45个重大项目，引进一批为项目审批服务的技术类中介机构，集聚发展高端生产和生活服务等新兴业态，做大总部经济、楼宇经济、街区经济，确保顺利通过国家中期评估。组建大数据发展促进中心，推进大数据产业园、铜官数谷建设，培育电商龙头企业，促进信息传输、电商产业发展。加快铜文化创意产业园、枞阳方氏名人文化园、南泉禅文化中心建设，打造区域特色文化产业集群。创建天井湖国家旅游度假区、普济圩国家现代农业庄园，推动浮山景区、大通古镇创建5A级景区，支持枞阳创建全域旅游示范区。改造提升传统商圈，打造商业特色街区，促进消费转型升级。

　　（二）激发创新创造活力，加快转换发展动能

　　提升自主创新能力。推动以科技创新为核心的全面创新，提高全社会研发投入强度，对标创建国家创新型试点城市。强化企业创新主体地位，引进培育一批高成长性创新型企业，新认定国家高新技术企业60家以上。加快创新平台建设，推进铜产业技术研究院、国家级铜基新材料及电磁线研发机构创建工作，全年新增省级以上各类研发机构不少于6家。组织实施10个省、市重大科技专项，争取集成电路封测装备等成为省级重大工程。深化产学研合作，扩大科技型企业贷款风险补偿资金池规模，推行科技创新券，积极筹建高校科技成果产业园，促进科技成果深度转化。发展一批众创空间，完善梯级孵化体系，引进10个以上高层次人才团队。加强知识产权创造、保护、运用，发明专利授权量增长10%以上，建设知识产权强市。

　　增强县区园区发展动力。全面落实促进县区经济发展政策，规范各级财权事权划分，强化县区经济社会管理权限，充分调动县区积极性，增强自主发展的主体意识。加大财政专项支持力度，引导县区集聚发展战略性新兴产业和特色产业，建设产业集群镇。增强县区投融资能力，支持县区整合存量资产资源，通过产业基金、总部基地、产业链整合、信用建设合作等模式，吸引社会资本参与产业培育发展和城镇建设。深化开发园区管理体制改革，有序推进开发园区优化整合，实行一县一区、一区多园，进一步推动各开发园区聚焦重点产业、聚焦发展主业。支持市经开区争创国家产城融合示范区，支持狮子山国家高新区打造区域创新高地，支持有条件的开发区创建省级高新区，推进“圈区管理”园区转型发展。完善县区、园区发展考核激励机制，努力形成争先进位、竞相发展的生动局面。

　　持续扩大有效投入。落实“四督四保”制度，提质提效建设项目，切实发挥投资对优化供给结构的关键性作用。紧扣投资质量效益，紧跟上级政策导向，紧盯重点产业发展路线图，紧贴经济社会发展迫切需要，加大项目谋划储备力度，争取更多项目纳入国家和省计划盘子，促进更多项目加快转化落地。全年实施亿元以上重点项目500个，计划投资370亿元。新开工建设年产400万张铝基覆铜板等111个重点项目，建成有色“奥炉”、高精度超薄电子铜箔等92个重点工程。落实促进民间投资政策，构建亲清新型政商关系，在基础设施、生态环保和社会公共服务等适宜领域，审慎遴选和推行一批PPP合作项目，激发民间投资活力。

　　提高对外开放绩效。坚定不移把“双招双引”作为首位工程，瞄准苏浙沪、珠三角等重点地区，发扬“钉钉子”精神，定向对接世界500强、央企和知名民企，精准实施产业链招商、板块招商、专业招商和以商引商，以存量引增量，以增量活存量。坚持招大引强、招新引优，每个县区、园区引进20亿元以上重大项目不少于1个，市经开区不低于3个。全年新引进亿元以上省外投资项目145个，到位资金550亿元，实际利用外资2.8亿美元。积极培育外贸主体与外贸增长点，大力发展服务贸易，促进加工贸易转型升级。加快建设皖中南B型保税物流中心，增强进境水果指定口岸功能，创造条件申报铜期货交割仓库。发挥港投集团作用，推进民营港口并购，启动江北港新区建设，完善港口集疏运体系，加快发展港口经济。

　　全面释放改革红利。持续加大简政放权力度，改革商事制度，推行“多证合一”“证照分离”试点，完善企业登记全程电子化；不折不扣落实减税降费政策，严格收费清单管理，实现清单之外无收费；巩固扩大“多规合一”改革成果，压缩建设项目总审批时限至50个工作日；深入落实部门监管清单制度，完善“双随机、一公开”监管机制。深化国资国企改革，完善铜化集团混合所有制改革，推进国企职工家属区“三供一业”分离移交，促进国有资产保值增值。

　　（三）大力推进城市建设，增强综合承载能力

　　强化规划引领。落实主体功能区战略，严格实施新一轮城市总体规划，推进城乡规划改革，出台规划管理条例，实现市区控制性详规和乡村规划全覆盖。

　　推进重大基础设施建设。全面落实推进跨江联动发展实施意见，加快补齐江北地区基础设施短板。推进大交通建设。做好铜枞宜城际、江北港铁路专用线、胥坝夹江大桥、国道330和青通河大桥等重大项目前期工作，争取动工兴建G3长江公铁大桥。升级区域路网，推进德上高速枞阳段建设，开工建设国道237和省道232、217，实现国道347枞阳段建成通车，国道236和省道221、335基本建成，省道320灾毁重建工程贯通。加强水利设施建设。配合实施引江济淮工程，落实长江铜陵段综合治理工程报批工作，完成长江干流崩岸应急治理任务，推进枞阳县城区防洪保安工程，加快顺安河三级航道整治进程。实施总投资20亿元的水利薄弱环节治理工程，完成中小河流治理项目5个，除险加固水库24座，建成城乡排涝泵站5个，进一步完善城乡防洪除涝体系。

　　提升城市功能品质。完善城市路网，建设义安大道上跨桥，打通翠湖二路东延伸段等一批断头路、丁字路，改造一批微循环道路。加快推进地下综合管廊建设，改造建设供水管网30公里、供气管道45公里，开工建设一批立体停车设施和电动汽车充电桩。优先发展公共交通，新增清洁能源公交车160台，开通市区至枞阳公交线路，成为全省公交示范市。启动大铜官山公园核心区建设，推进街头绿地游园建设，提升城市景观。强力组织实施“两治三改”行动，清理违法用地700亩以上，拆除违法建设14万平方米，改造棚户区2100户、农村危房1600户，完成10个老旧小区整治。建设新型智慧城市，超前谋划新一代互联网建设，加快谋划布局5G网络，着力引进高端云建设主体，推进大数据应用，建设城市物联网，加快建设“城市大脑”，引领铜陵进入智联时代。加强城市管理，着力解决交通拥堵及停车难、停车乱等难题，提升网格化、精细化管理水平。

　　（四）实施乡村振兴战略，提升“三农”工作水平

　　努力让现代农业“强”起来。深化农业供给侧结构性改革，坚持质量兴农、绿色兴农，促进农业由增产向提质转变，推动枞阳由农业大县向经济强县迈进。划定粮食生产功能区和重要农产品生产保护区，新增高标准农田5.78万亩，稳定粮食产量。推进种植业、畜牧业、渔业、农产品加工流通业转型升级和融合发展，加快水稻、牡丹、食用菌全产业链开发，提高农业产业化发展水平。积极推广现代农业技术和科技成果，建设一批标准化养殖示范区，新增“三品一标”农产品15个以上。积极培育新型农业经营主体，促进小农户和现代农业发展有机衔接，新增市级龙头企业10个、示范家庭农场100家、现代农业产业化联合体5个。

　　努力让农村资产“活”起来。深化农村土地制度改革，加强农村土地确权登记颁证成果应用，完善承包地“三权”分置制度，进一步盘活用好农民闲置宅基地和闲置农房。深化农村集体产权制度改革，全面开展农村集体资产清产核资，加快推进农村集体资产股份合作制改革试点，推动农村“三变”改革试点取得实质性进展。

　　努力让乡村环境“美”起来。进一步培育大通、钟鸣和铜都创新、西湖文体等特色小镇，完善建设机制，提升产业层次，促进健康发展。扎实推进美丽宜居乡村建设，建成省级中心村23个、市级中心村16个、特色自然村6个。加强传统村落保护，创建一批人居环境建设和休闲旅游示范村。综合整治农村人居环境，一体化推进农村环境“三大革命”，完成3.4万常住农户卫生厕所改造，实现乡镇政府驻地生活污水处理设施全覆盖。启动实施农村道路提升工程三年行动计划，建好、管好、护好、运营好农村公路。实施农村饮水安全巩固提升和新一轮农村电网改造升级工程，持续改善农村基础设施。

　　（五）坚决打赢污染防治攻坚战，加快建设美好家园

　　实施生态环保优先战略。把生态环保贯穿于经济社会发展各方面和全过程，空间上守住生态红线保护底线，行业上守住排放总量底线，项目上守住环境准入标准底线。全面完成污染源普查，组织实施污染防治突击与攻坚，依法严惩环境违法行为，推动生态环境质量根本好转，打造有土皆绿、是水则清、四季花香、处处鸟鸣的美丽铜陵。

　　重拳整治环境突出问题。坚持对标对表，不折不扣落实中办调研回访、中央和省环保督察反馈问题整改任务，巩固发展整治成果。打赢蓝天保卫战，实施重点行业涉气治理改造项目200个，深入推进“散乱污”企业整治，有效控制建筑工地、露天物料堆场等重点区域扬尘污染，强化机动车污染防治，严格落实秸秆禁烧等污染防治责任，进一步明显降低PM2.5浓度，明显减少重污染天数，明显增强人民群众的蓝天幸福感。打好碧水攻坚战，全面落实“河长制”“湖长制”，推进长江流域环境整治，加强城市饮用水水源地保护和枞阳县城备用水源地建设，建成运营狮子山生活污水处理厂，提标改建新民、西湖污水处理厂，加快建设雨污分流管网，完成惠溪河、狮矿大沟黑臭水体整治，为群众提供更多亲水环境。打好净土持久战，深入开展土壤污染状况详查，推进土壤污染治理与修复试点，加强重金属污染综合防治，加大固体废弃物处理和农业面源污染防治力度，保障农产品质量和人居环境安全。

　　深入推进绿色发展。继续实施发展循环经济行动计划，大力推进清洁生产，建成南部城区集中供热项目，推进城市能源管控中心建设，加快金桥开发区国家级园区循环化改造，争创国家资源循环利用基地。设立绿色发展基金，支持节能环保、清洁能源等产业发展。推进秸秆回收、综合利用，秸秆综合利用率达87%以上。坚持节约集约用地，清理闲置土地不少于1000亩，争创全国国土资源节约集约模范市。积极参与国家节水行动，创建国家节水型城市。

　　强化生态保护修复。谋划实施长江防护林、滨水生态廊道等重大生态工程，筑牢生态安全屏障。落实淡水豚国家自然保护区勘界工作，严格实施全面禁渔、船舶限速等保护措施。启动大规模国土绿化行动，持续推进绿化提升工程，完成人工造林1万亩，保护培育森林生态系统，全面推行“林长制”。编制湿地保护工程规划，实施西湖湿地修复保护二期工程。落实生态补偿机制，加快废弃矿区地质环境和生态修复治理，推进绿色矿山建设。

　　（六）夺取脱贫攻坚新胜利，提高人民群众获得感

　　强力组织脱贫攻坚。坚持把脱贫攻坚作为重大政治任务和最大民生工程，举全市之力，集全市之智，支持枞阳脱贫攻坚，确保今年33个贫困村出列、3万贫困人口脱贫，努力实现“三战三捷”。坚持精准扶贫、精准脱贫，完善包保机制，严格责任落实，确保脱贫质量。深入实施脱贫攻坚“十大工程”，大力推行“四带一自”模式，落实教育扶贫、健康扶贫等各项政策，加强基层基本公共服务功能建设，创新推进保险扶贫，建立健全稳定脱贫长效机制。扎实开展“脱贫攻坚作风建设年”活动，加强扶贫工作队管理，以过硬作风打赢脱贫攻坚硬仗。

　　织密扎牢民生保障网。聚焦“七有”领域，动态实施38项民生工程，新增加“学前教育经费保障”“洲圩区渡口标准化建设”等内容。支持枞阳县全面推进农村自来水并网工程，提高江北地区农村饮水质量。

　　办好公平优质教育。擦亮铜陵均衡教育金字招牌，实施优质教育扩面工程，推进普惠性幼儿园建设，全面完成薄弱学校改造工程，着力解决中小学生课外负担重等突出问题，解决好婴幼儿照护和儿童早期教育服务问题。推进高中阶段学校考试招生制度改革，普及高中阶段教育。切实保障特殊群体受教育权益。深化产教融合、校企合作，推动铜陵职业技术学院、安工学院和技师学院发展，创建省级示范性公共实训基地。支持铜陵学院加快建设地方应用型高水平大学。支持规范社会力量兴办教育。加强师资培养培训，建设高素质教师队伍。

　　加强就业和社会保障工作。坚持就业优先战略，鼓励创业带动就业，扎实做好农民工、去产能分流职工、高校毕业生等群体就业和退役军人安置工作，确保零就业家庭动态清零。全面实施全民参保计划，完善统一的城乡居民基本医保，稳步提升城镇职工、城乡居民及被征地农民养老金标准。统筹城乡救助体系，试点大病商业补充保险，提高城乡低保、特困人员供养标准，巩固提升“救急难”试点市建设成果。

　　推动文化事业繁荣发展。完善公共文化服务网络，构建全民阅读、全民艺术普及体系，广泛开展群众性文化活动，确保公共文化服务体系示范区建设顺利通过国家验收。着力打造中国铜博物馆，筹建市美术馆新馆，完成数字图书馆提升工程，推进基层综合文化中心建设，努力提高公众科学文化素质，促进城乡公共文化均衡发展。繁荣发展哲学社会科学，加强党史、地方志和档案工作。

　　推进健康铜陵建设。深化公立医院改革，推进医联体建设，提高基层医疗卫生机构服务能力。加强计划生育管理和服务，培育壮大健康产业。深入开展爱国卫生运动，确保国家卫生城市通过复审。推进全国居家和社区养老服务改革试点，扩大医养结合试点范围，推动老龄事业和老龄产业协调发展。广泛开展全民健身活动，推进体教结合，提升青少年身体素质。

　　（七）切实防范化解重大风险，维护社会和谐稳定

　　加强地方金融与债务风险防控。深化金融监管体制改革，组建地方金融监管机构，落实市县（区）政府金融监管责任、金融机构防范风险主体责任，建立健全风险监测预警、早期干预和应急处置机制，严守不发生系统性区域性金融风险底线。加强重点行业、企业信贷风险排查防控，支持银行化解不良资产风险，加大金融领域违法失信行为联合惩戒力度，坚决打击恶意逃废债行为。大力整顿规范金融秩序，坚决打击违法违规金融活动，持续加强互联网金融监管。组织开展“四清四实”专项整治，打好政府隐性债务清理战、存量稳妥处置战、增量严控战，加强债务预算管理和限额管理，加快存量债务置换步伐，从严把关各类政府性投资项目，优化债务结构，严控债务规模，确保政府债务风险可控。

　　加强公共安全防控。深化“平安铜陵”建设，完善立体化、信息化社会治安防控体系，依法打击和惩治黄赌毒黑拐骗等违法犯罪活动，保护人民群众人身权、财产权、人格权。加强食品药品全过程监管，实现基层监管所和大型食用农产品批发市场快检体系全覆盖。深入开展安全生产“铸安”行动，推进道路交通、消防、危险化学品等重点领域安全防控监测信息系统建设，确保不发生较大以上安全生产事故。开展地质灾害防御行动，强化地质灾害应急处置和隐患点治理。发展气象、水文、地震等事业，提升防灾减灾救灾能力。加强网络安全管理，严厉打击网上虚假信息诈骗、倒卖个人信息等不法行为，营造清朗网络空间。

　　加强社会治理工作。健全基层社会治理体系，推进智慧社区建设，完善网格化管理，激发社会组织参与社会治理积极性，完成村委会、社区居委会换届选举任务，推动政府治理和社会调节、居民自治良性互动。完善社会矛盾排查预警和调处机制，扎实化解信访积案，畅通网上信访主渠道，推动信访工作重心下移、关口前移，下大力气解决一批历史遗留涉稳问题。大力推进“七五”普法，落实“谁执法、谁普法”工作责任制。加强对老人、农村留守儿童的关爱服务，切实维护妇女、儿童权益。有序实施残疾人两项补贴，推进社会福利和慈善事业发展。加强全民国防动员、国防教育、人民防空和拥军优属工作，努力实现全国双拥模范城“五连冠”。

　　四、全面加强政府自身建设

　　跨入新时代，发展形势逼人；接力新征程，政府重任在肩。我们必须顺应时势、奋发向上，知难不畏难、抢滩不守滩、扛事不避事，立大志、担大任、吃大苦、创大业，努力打开新局面，展现新作为。

　　（一）强化政治建设，打造忠诚履职政府。坚持用习近平新时代中国特色社会主义思想武装头脑、统一行动，坚定理想信念，牢记根本宗旨。严守政治纪律、政治规矩，牢固树立“四个意识”，坚决维护党中央权威和集中统一领导，始终在政治立场、政治方向、政治原则、政治道路上，同以习近平同志为核心的党中央保持高度一致，坚决做到“五个纯粹”。推进“两学一做”学习教育常态化制度化，扎实开展“不忘初心、牢记使命”主题教育，不断加强党性锻炼，确保始终同全市人民想在一起、干在一起。

　　（二）严格依法行政，打造民主法治政府。推动政府系统工作人员自觉尊法学法守法用法，严格遵循法定权限和程序履职尽责，自觉运用法治思维和法治方式推动工作。加强政府立法工作，严格决策程序，完善合法性审查提前介入机制，基本实现村（居）委会法律顾问全覆盖。深化行政执法体制改革，严格落实重大执法决定法制审核制度，完善行政执法与监察司法联动衔接机制，推进部门之间执法监管信息共享。自觉接受人大法律监督、工作监督和政协民主监督，认真办理人大代表建议和政协提案。自觉发展协商民主，广泛听取各民主党派、工商联和无党派人士意见，发挥工会、共青团、妇联等群团组织联系群众的桥梁和纽带作用。完善政府内部层级监督和专门监督，全面推进政务公开，让政府权力在阳光下运行。

　　（三）强化责任落实，打造务实担当政府。负责才有担当，落实方见本领。我们要立足当前，着眼长远，坚持求真务实，不提不切实际的目标，不搞寅吃卯粮的工程；立足于稳，着眼于进，该稳的要稳住，该进的要进取，倡导干字当头，始终保持真抓的实劲、敢抓的狠劲、善抓的巧劲、常抓的韧劲；立足创优，着眼争先，追求极品极致，绝不停留于“一般化”、满足于“还可以”、止步于“中上等”。牢固树立一盘棋思想，执行决策、落实部署，提倡“马上就办”，第一时间作出反应，第一时间跟进落实，第一时间取得实效。坚持责任倒逼、问题倒逼、目标倒逼、督查倒逼，经常抓、抓经常，反复抓、抓反复，一以贯之、一抓到底。优化考核指标体系，严格绩效考核，完善落实正向激励机制、容错纠错机制，为坚定者、奋进者、搏击者撑腰鼓劲，营造竞相负责、主动作为的浓厚氛围。

　　（四）增强执行能力，打造高效服务政府。各级政府特别是领导干部都要增强能力危机感、本领恐慌感。要找准能力短板、本领弱项，有针对性地开展大学习、大培训，丰富知识储备、完善知识结构，培养专业能力、弘扬专业精神，不断增强推动高质量发展的本领。要大兴调查研究之风，推动干部深入到困难地区和工作推不开的地方，到改革开放的主战场、维护稳定的第一线、服务群众的最前沿，在干事中长本事，在历练中变“老练”。适应时代发展要求，建立健全大数据辅助决策和治理机制，推进政府管理创新。完善“互联网+政务服务”体系，建成市县乡村四级一体化政务服务平台，推进一网通办，推行“最多跑一次”“不见面审批”，让群众办事更便捷、政务服务更智慧。

　　（五）坚持正风肃纪，打造清正廉洁政府。坚持问题导向、严字当头，以“永远在路上”的韧劲，扛起从严治党主体责任。严格贯彻中央八项规定精神和省、市委实施细则，不断加强党内政治生活、政治文化、政治生态建设。深化廉政风险防控，深入推进审计监督全覆盖，加强对财政资金使用、公共资源交易、国有资产管理等重点领域监督管理，严防权力滥用和设租寻租，坚决把权力关进制度的笼子。坚持整治“四风”不止步，坚持反腐败无禁区、全覆盖、零容忍，坚决查处发生在群众身边的“四风”和腐败问题，坚决查办违纪违法案件，巩固发展反腐败斗争压倒性态势。政府各级领导干部要带头执行廉洁自律准则，认真履行“一岗双责”，发挥示范作用，形成“头雁效应”，进一步建设好政治生态的绿水青山。

　　各位代表！

　　鲲鹏展翅凌万里，逐梦扬帆再起航。让我们紧密团结在以习近平同志为核心的党中央周围，在省委、省政府和中共铜陵市委的坚强领导下，不忘初心、牢记使命，同心同德、顽强拼搏，为奋力开创新时代转型发展新局面，加快建设现代化幸福美丽新铜陵而不懈奋斗！
